# Biliousness

**Published:** 1872-05

**Authors:** 


### HALL'S

### JOURNAL OF HEALTH.

Vol. XIX.—MAY, 1872.—No. V.

### BILIOUSNESS

Means that the bile, which in reality is the waste of the system, the washings out of its used and useless particles, has not been separated from the blood, and remains in it, keeps it dark, thick, impure, poisonous, and unfit to carry on the life processes; this blood, being thick and heavy, is sluggish, does not pass along the blood-vessels freely, but clogs them, dams them up, becomes impacted in them, congested; this congestion making itself felt most in those parts of the system which have been weakened, either by accident, disease, or hereditarily.

It is the business of the liver to withdraw from the blood those useless particles, which, being mixed together, make what is called bile; these particles are naturally conveyed from the liver into the gall-bladder, out of which it passes, drop by drop, into the top or beginning of the bowels, near the point where the food passes from the stomach into this same top of the bowels, and is especially active after meals. It is a strikingly beautiful exhibition of the economy of nature, that this bile, which is perfectly useless to the body, as waste matter, is made to have a purgative effect on the bowels, stimulating them to pass from them the refuse of food which contained no nutriment. The Creator could as easily have added another apparatus to the machinery of man which would have had a specific effect, the same effect as the bile has; but that would have made the machine more complicated; and He preferred to make nothingness useful; He preferred to order the adaptations so that a useless thing should do a useful work, a work absolutely necessary to the well being and safety of the body; for we all know that if the bowels cease to act, we certainly die. This is of a piece with the divine operation in the moral world; worthless men, wicked men, are made use of, to forward his great purposes, as clearly expressed in the Holy Record, "making the wrath of man to praise Him."

The effect of this presence of bile in the blood, of this congestion, compaction, is to clog up the whole machinery of the body, like a clock that is so dirty it scarcely runs at all; the limbs don't work, and the man feels indisposed to do anything; the brain does not work, and the bilious man can't think to advantage; his head is heavy, his eyes are dull, there is no life, no animation, no appetite; and instead, a universal feeling of discomfort; the feet are cold, the head aches, the bowels costive; in short, the whole mental, moral, and physical nature is in a state of demoralization. Now for the remedy:—

First, cut off the supply of blood, for there is too much in the body already, and as the blood is made out of the food eaten, if nothing is eaten for a day or two, half the work is done, and eventually the whole, by continuing to eat but little, thrice a day for a short time, until the symptoms have disappeared. This is the doctors' method.

Another is, stimulate the liver; make it work, by taking such things as are known to "act" on the liver; we don't know why they should act on the liver, or how, but we know they do do it. One thing acts on the brain, as whiskey; another acts on the stomach, as tartar-emetic; a third acts on the bowels, as a dose of salts; so there are medicines which "act on the liver;" this is the quickest way, and the laziest way, and the least self-denying way of getting rid of biliousness; but it is not the best way; it is the worst way. So no information will be given here as to what medicines would best act on the liver; for then, when a man became bilious, he would take a dose of physic, and in a short time he would be all the time dosing. Besides, after a while all remedies begin to lose their legitimate effect, and either become useless in any quantity, or must be taken in large quantities, so large sometimes as to become poisonous and fatal.

There are, however, two things which we can take for biliousness, and either of them will cure in all curable cases; but they won't be taken; hence no harm will result from imparting information.

Blood, like many other liquids, is thinned by being warmed; hence warm up the blood. Nature teaches this, for bilious persons are very chilly. One way is to

TAKE A SWEAT, a good, old-fashioned sweat, by getting into bed with hot bricks to the feet, and bottles of hot water under the arms, and a quart or two of hot catnip tea, or any other kind of tea, although hot water will do quite as much good, only it is not so pleasant to take; ginger tea, red pepper tea, all have their virtues, but the most virtue is in the heat of the fluid swallowed; then have yourself tucked in all around in a feather bed, so as to have the sweat roll out of you in great big wholesome drops for an hour or two; repeat this daily until well; eating in the meanwhile, bread, fruit, soups with bread crust in them; this treatment not only renders the blood more fluid, so that it will circulate more easily, but it diminishes the bulk of the blood, the weight of it, for the sweat comes out of the blood, being its more watery particles. But after a "sweat," the person should cool off slowly, gradually, in the course of an hour.

But there is another plan and a better: "Take exercise."

The best is steady work in the open air, as in ploughing, chopping wood, splitting rails, threshing grain, etc., but if you are so unfortunate as to have nothing to do, no useful work to perform, row a boat, or take a steady walk of an hour or two's duration; a walk with a pleasurable result at the end of it; a walk involving such physical activity as to cause a gentle and general perspiration, and such mental or moral energy as to cause self-forgetfulness, and an entire absorption in the thing in hand. This interested, pleasurable exercise not only does all the good of the sweating operation, but has an additional power in diminishing the quantity of the blood, in working the waste particles out of the system, by the friction of the multitude of muscles which are employed in walking. If, in addition to the exercise of walking, there is brought into requisition various bendings and twistings of the body, all these have the effect to stimulate the liver, by pressing upon it, like pressing water out of a sponge; because it is a large, soft body, weighing several pounds, at the left side, above the lower edge of the ribs.

Hence it is that persons, even of the bilious temperament, who are on their feet for a great part of daylight, stooping down, straightening up, pushing, pulling, and lifting, are seldom troubled with "biliousness," unless they take cold. There are many ladies in New York who keep off biliousness, or get rid of it when an accidental cold brings it on, or close confinement to the house for several days, by taking an active walk of an hour or more every day, in marketing, shopping, calling upon friends, or making visits of charity and good-will to the sick, and suffering, and unfortunate. Who does not see the wonderful wisdom and beneficence of the great Creator of us all; who punishes to save, who brings blessings out of cursing? for in that He said, "In the sweat of thy face shalt thou eat bread," He so arranged it that the very labor which man was destined to perform, would by the very sweatings keep the human system in a healthy condition, making labor a pleasure and a means of glorious good health.

